# Sampling the Solution Space in Genome-Scale Metabolic Networks Reveals Transcriptional Regulation in Key Enzymes

**DOI:** 10.1371/journal.pcbi.1000859

**Published:** 2010-07-15

**Authors:** Sergio Bordel, Rasmus Agren, Jens Nielsen

**Affiliations:** Systems Biology, Department of Chemical and Biological Engineering, Chalmers University of Technology, Gothenburg, Sweden; University of Wisconsin-Madison, United States of America

## Abstract

Genome-scale metabolic models are available for an increasing number of organisms and can be used to define the region of feasible metabolic flux distributions. In this work we use as constraints a small set of experimental metabolic fluxes, which reduces the region of feasible metabolic states. Once the region of feasible flux distributions has been defined, a set of possible flux distributions is obtained by random sampling and the averages and standard deviations for each of the metabolic fluxes in the genome-scale model are calculated. These values allow estimation of the significance of change for each reaction rate between different conditions and comparison of it with the significance of change in gene transcription for the corresponding enzymes. The comparison of flux change and gene expression allows identification of enzymes showing a significant correlation between flux change and expression change (transcriptional regulation) as well as reactions whose flux change is likely to be driven only by changes in the metabolite concentrations (metabolic regulation). The changes due to growth on four different carbon sources and as a consequence of five gene deletions were analyzed for *Saccharomyces cerevisiae*. The enzymes with transcriptional regulation showed enrichment in certain transcription factors. This has not been previously reported. The information provided by the presented method could guide the discovery of new metabolic engineering strategies or the identification of drug targets for treatment of metabolic diseases.

## Author Summary

The sequencing of full genomes and the development of high-throughput analysis technologies have made available both genome-scale metabolic networks and simultaneous transcription data for all the genes of an organism. Genome-scale metabolic models, with the assumption of steady state for the internal metabolites, allow the definition of a region of feasible metabolic flux distributions. This space of solutions can be further constrained using experimental flux measurements (normally production or uptake rates of external compounds). Here a random sampling method was used to obtain average values and standard deviations for all the reaction rates in a genome-scale model. These values were used to quantify the significance of changes in metabolic fluxes between different conditions. The significance in flux changes can be compared to the changes in gene transcription of the corresponding enzymes. Our method allowed for identification of specific reactions that are transcriptionally regulated, and we further identified that these reactions can be ascribed to a few key transcription factors. This suggests that the regulation of metabolism has evolved to contain a few flux-regulating transcription factors that could be the target for genetic manipulations in order to redirect fluxes.

## Introduction

Systems Biology aims to use mathematical models to integrate different kinds of data in order to achieve a global understanding of cellular functions. The data to be integrated differ both in their nature and measurability. The availability of DNA microarrays allows for the comparative analysis or mRNA levels between different strains and conditions. These data provide genome-wide information, and changes in expression at different conditions are expressed in statistical terms such as p-values or Z-scores that quantify the level of significance in transcriptional changes. The availability of annotated genome-scale metabolic networks allowed mapping of the transcriptional changes in metabolic genes on to their corresponding metabolic pathways and defining significantly up or down regulated sub-networks [1]. Even though this allows for identification of transcriptional hot-spots in metabolism, this does still not provide information about whether there are any changes in metabolic fluxes in these pathways, as it has been shown that in general there is no clear correlation between gene expression and protein concentration [2] or metabolic flux [3], [4].

Metabolic fluxes are the result of a complex interplay between enzyme kinetics, metabolite concentrations, gene expression and translational regulation. Metabolic fluxes can be directly measured using ^13^C labeling experiments [5]. However, flux data obtained using this approach differ from gene expression data in two main features: 1) their determination is only possible for a relatively small subset of all the reactions in a genome-scale metabolic network and 2) they are indirect data in the sense that the fluxes are quantified obtained by fitting measured labeling patterns using a simple metabolic model. The complexity of the mRNA-flux dependence and the disparity in the nature of both kinds of data make their integration an important challenge.

In this paper we propose a method to integrate gene expression data with flux data by transforming a limited amount of quantitative flux data into a genome-scale set of statistical scores similar to the one obtained from DNA microarrays. In order to do that, a set of experimental exchange fluxes are fixed for each of the studied conditions or for each of the strains investigated, and a sampling algorithm is then used to obtain a set of flux distributions satisfying the experimental values. This approach allows for obtaining means and standard deviations for each flux in the genome-scale network. From the mean and standard deviation it is possible to derive statistical scores for the significance of flux change between conditions [6], [7]. Random sampling in the region of feasible flux distributions has been previously used to study the statistical distribution of flux values and determine a flux backbone of reactions carrying high fluxes [8] as well as to define modules of reactions whose fluxes are positively correlated [9], [10]. Also mitochondria related diseases have been analyzed using random sampling [11]. All the works published so far used the Hit and Run algorithm to perform the sampling [7].

**Figure 1:**
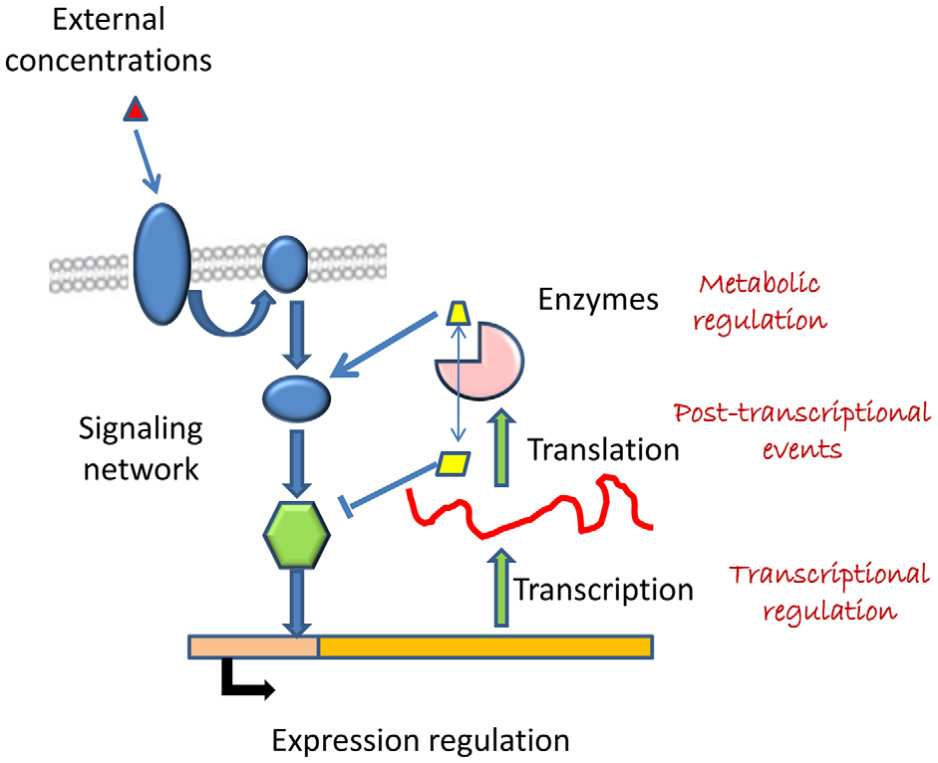
Illustration of the regulatory mechanisms of cellular metabolism. The fluxes can be regulated at the level of mRNA transcription, by the concentrations of the metabolites or by intermediate steps such as translation or activation of the enzymes.

By dividing the average difference among two conditions (e.g. carbon sources or mutant strains) by its standard deviation, it is possible to obtain Z scores for each metabolic flux. These scores can be transformed into p-values that measure the significance of change of each flux (see methods). By comparing these p-values with the p-values derived from gene-expression arrays, the enzymes in the network can be classified as: 1) enzymes that have a significantly correlated change both in flux and expression level (reactions showing transcriptional regulation), 2) enzymes that show a significant change in expression but not in flux (we will refer to them as showing post-transcriptional regulation) and 3) enzymes that show significant changes in flux but not a change in expression (metabolic regulation). Hereby we provide a framework that allows for global classification of reaction fluxes into those that are transcriptionally regulated, post-transcriptionally regulated and metabolically regulated (see Fig. 1). This will have substantial impact on the field of metabolic engineering where changes in gene-expression are often used as the key means to alter metabolic fluxes. In the paper we show the use of the presented framework for the analysis of the yeast *Saccharomyces cerevisiae* grown at different growth conditions and for the analysis of different deletion mutants.

The combined use of random sampling of genome-scale metabolic networks and expression data allows for global mapping of reactions that are either transcriptionally or metabolically regulated. This information can be used to guide the engineering of microbial strains or as a diagnosis tool for studying metabolic diseases in humans. In particular we should highlight that reactions in which there is no relation between gene transcription level and metabolic flux are not suitable targets for flux increase via gene over-expression. Through analysis of different data sets the method revealed that many changes in gene expression are not correlated with a corresponding change in metabolic fluxes. The use of gene-expression data alone can therefore be misleading. However, our method allowed for identification of many specific reactions that are indeed transcriptionally regulated, and we further identified that the expression of these enzymes is regulated a few key transcription factors. This fact suggests that the regulation of metabolism has evolved to contain a few flux-regulating transcription factors that could be the target for genetic manipulations in order to redirect fluxes.

## Results/Discussion

### Comparisons between different sampling methods

**Figure 2:**
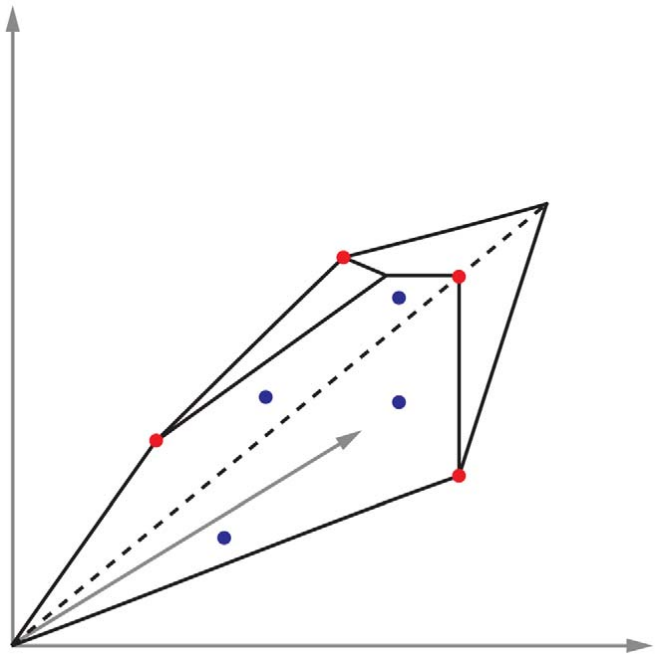
The red points illustrate the sampling in the corners of the region of allowed solutions. The blue points illustrate the uniform random sampling inside the space of allowed solutions.

Here we propose a sampling method that finds extreme solutions among the feasible flux distributions of the metabolic network. These solutions correspond to the corners in the region of allowed flux distributions, and in mathematical terms they are elements of the convex basis of the region of feasible solutions (which is a convex set). The COBRA Toolbox [12] includes a random sampling option that uses the Hit and Run algorithm [13] to obtain points uniformly distributed in the region of allowed solutions. The difference between the two sampling methods is illustrated in Fig. 2.

In order to assess the accuracy of our sampling method to estimate the average fluxes and their standard deviations, we compared a set of internal fluxes measured with ^13^C labeling [14] with predictions using 500 sampling points obtained using the sampling method in the convex basis and 500 sampling points obtained using the sampling algorithm implemented in the COBRA Toolbox. The results are summarized in Table 1 where our method is labeled Convex Basis (CB), because it samples elements of the convex basis of the region of allowed solutions (see above), and the method from the COBRA Toolbox is labeled Hit and Run (HR). The Z values in the table are the number of standard deviations that the real value is deviating from the calculated mean.

**Table 1:** Real and estimated fluxes in *S. cerevisiae* at aerobic and anaerobic growth conditions.

Aerobic conditions	Flux	Mean (HR)	Variance (HR)	Mean (CB)	Variance (CB)	Z (HR)	Z (CB)
**Fructose-bisphosphate aldolase**	0.70	0.76	0.0001	0.73	0.0098	3.38	0.19
**Pyruvate kinase**	1.50	1.49	0.0351	1.43	0.344	0.024	0.12
**Pyruvate dehydrogenase**	0.47	1.03	0.0004	0.98	0.015	28	4.14
**Citrate synthase**	0.71	0.99	0.0178	0.94	0.1868	2.08	0.53
**Pyruvate carboxylase**	0.32	0.23	0.0014	0.22	0.0124	2.44	0.96
**Glucose-6-phosphate 1-dehydrogenase**	0.52	0.37	0.0012	0.41	0.0881	4.25	0.37
**Pyruvate decarboxylase**	0.53	0.07	0.0004	0.08	0.0092	23	4.7

Anaerobic conditions							
**Fructose-bisphosphate aldolase**	5.82	5.62	0.0053	5.67	0.30	2.78	0.28
**Pyruvate kinase**	10.37	10.39	1.93	10.49	25.62	0.019	0.025
**Pyruvate dehydrogenase**	0.26	0.59	0.0114	0.87	0.45	3.16	0.91
**Citrate synthase**	0.19	0.96	0.0271	1.03	1.03	4.7	0.83
**Pyruvate carboxylase**	0.32	0.32	0.0261	0.88	2.82	0.028	0.33
**Glucose-6-phosphate 1-dehydrogenase**	0.32	1.35	0.0441	1.20	2.40	4.9	0.56
**Pyruvate decarboxylase**	9.54	9.82	1.71	9.21	2.20	0.22	0.22

HR refers to the Hit and Run algorithm and CB to the Convex Basis algorithm.

The means obtained by the two sampling methods are very similar for most of the reactions; however the standard deviations found using the HR algorithm are significantly smaller. With the HR method the real values for the fluxes in many cases deviate several standard deviations from the mean, A high value of Z indicates that the real value has a very low chance of being obtained using the considered sampling method (or in other words: the real value does not belong to the family of solutions that is generated by the sampling method). The number of samples with the HR algorithm was increased up to 5000 to check possible effects of the sample size on the standard deviation. Only small increases were observed for the standard deviations of the studied fluxes.

Using the CB algorithm we obtain higher standard deviations and the real flux is for most reactions less than one standard deviation away from the mean flux. We can therefore conclude that the CB sampling method gives more realistic standard deviations for the fluxes. This is important if we want to compare the significance of flux changes between conditions. An underestimated standard deviation would make some flux changes appear as being significant even though they may not be in reality, and our method therefore gives a more conservative list of significantly changed reaction fluxes than the HR algorithm.

### Comparisons between different carbon sources and mutant strains

To evaluate our method we used data for the yeast *S. cerevisiae*. Data from growth on four different carbon sources (glucose, maltose, ethanol and acetate) in chemostat cultures and five deletion mutants (grr1Δ, hxk2Δ, mig1Δ, mig1Δmig2Δ and gdh1Δ) grown in batch cultures were used. The exchange fluxes and gene expression data for the mentioned conditions have been published earlier [15]–[17].

**Figure 3:**
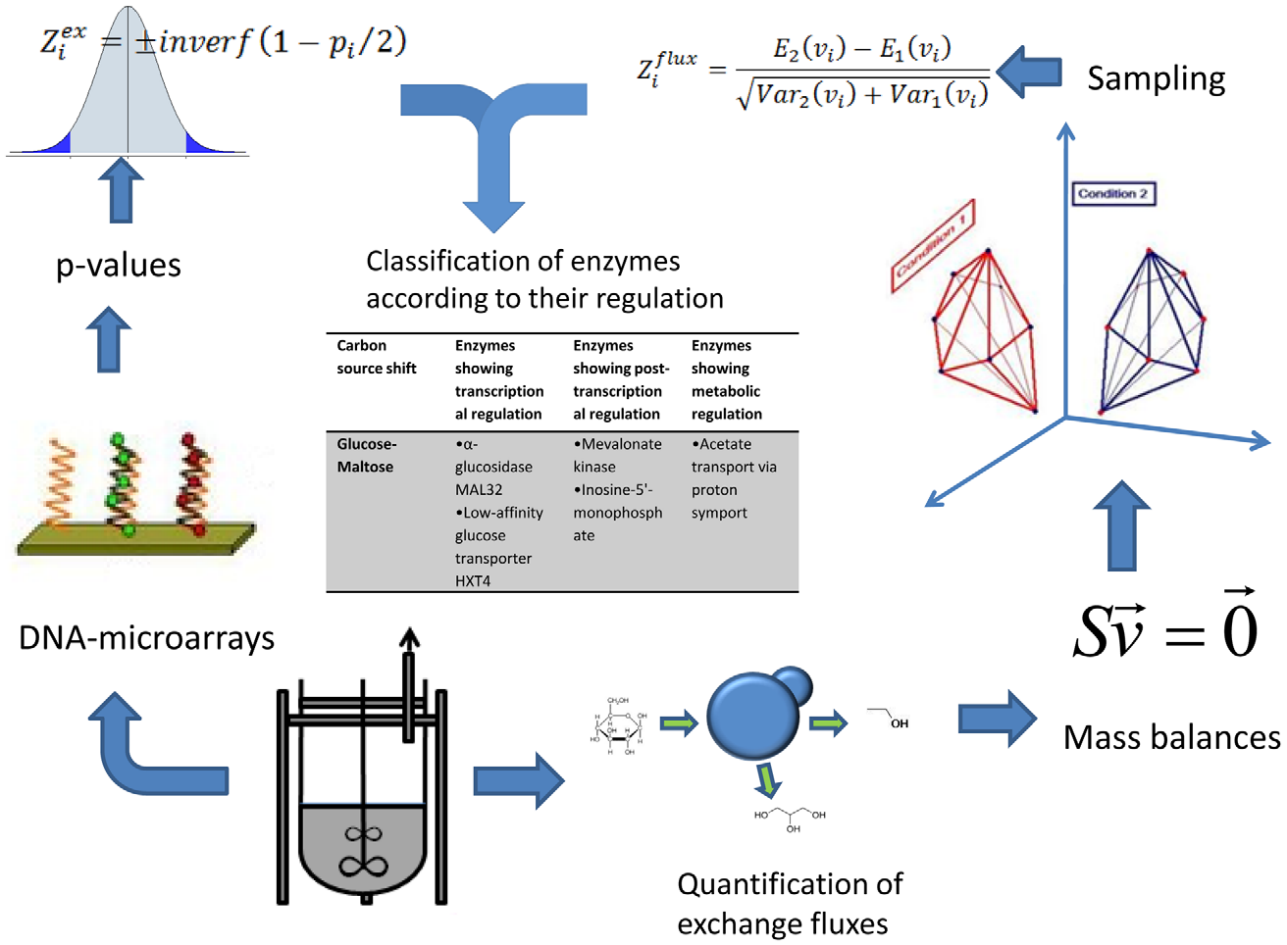
This figure illustrates the different steps of our method. Two kinds of data are extracted from fermentations, gene expression data and production and consumption rates of different metabolites. The gene expression data are transformed into significance scores and p-values for the expression change of the metabolic genes. The measured fluxes are used to constrain the solution spaces corresponding to different conditions. A sampling among the allowed solutions gives averages and standard deviations for each reaction rate in the metabolic network. These values can be obtained to obtain significance scores and p-values for the changes in reaction rates. The p-values for changes in expression and in reaction rates can be combined to obtain the probabilities for a correlated change between both values (transcriptional regulation), changes in rate not correlated to transcriptional changes (metabolic regulation) and changes in transcription that are not correlated to changes in rate (which we refer to as posttranscriptional regulation).

Our method obtains probability scores for each enzyme in the metabolic network (see methods) and this allowed us to classify the enzymes as transcriptionally regulated (correlation between flux and gene expression), post-transcriptionally regulated (changes in gene expression don't cause changes in flux) and metabolically regulated (changes in flux are not caused by changes in gene-expression). The cut-off chosen for this classification was a probability score above 0.95. Tables 2 and 3 show the 10 top scoring enzymes in each group (or fewer when less than 10 enzymes had a score exceeding 0.95). The method is illustrated in Fig. 3.

**Table 2:** Top scoring enzymes for transcriptional, post-transcriptional and metabolic regulation for changes in carbon source.

Carbon source shift	Enzymes showing transcriptional regulation	Enzymes showing post-transcriptional regulation	Enzymes showing metabolic regulation
**Glucose-Maltose**	• α-glucosidase MAL32	• Mevalonate kinase	• Acetate transport via proton symport
	• Low-affinity glucose transporter HXT4	• Inosine-5′-monophosphate dehydrogenase IMD2	
		• Asparagine synthetase 1	
		• (DL)-glycerol-3-phosphatase 1	
		• Uncharacterized deaminase	
		• Nicotinate-nucleotide pyrophosphorylase	
		• Mevalonate kinase	
		• Mevalonate kinase	
		• Glycerol-3-phosphate dehydrogenase [NAD+] 1	
**Glucose-Ethanol**	• Phosphoenolpyruvate carboxykinase	• Formate dehydrogenase 2	• Fructose-bisphosphate aldolase
	• Fructose-1,6-bisphosphatase	• ATP-NADH kinase	• Triosephosphate isomerase
	• Isocitrate lyase	• Sulfate permease 1	• Pyruvate dehydrogenase E1 component subunit alpha [m]
	• Malate dehydrogenase [c]	• Formate dehydrogenase 1	• Alpha-ketoglutarate dehydrogenase
	• Citrate synthase [p]	• Dicarboxylate transporter [m]	• Succinyl-CoA ligase [ADP-forming] subunit beta [m]
	• Ribose-5-phosphate isomerise	• NADP-specific glutamate dehydrogenase 2	• Malate synthase 2, glyoxysomal
	• Low-affinity glucose transporter HXT4	• Uncharacterized deaminase	• Glucose-6-phosphate 1-dehydrogenase
	• External NADH-ubiquinone oxidoreductase 2 [m]	• Probable 6-phosphogluconolactonase 3	• Cytochrome b-c1 complex subunit Rieske [m]
	• Glucose-6-phosphate isomerase	• 6-phosphofructo-2-kinase 2	• Adenylate kinase [c]
		• Nucleoside diphosphate kinase	
**Glucose-Acetate**	• Fumarate hydratase [m]	• Ribonucleoside-diphosphate reductase large chain 1	• Fructose-bisphosphate aldolase
	• Phosphoenolpyruvate carboxykinase [ATP]	• Phospho-2-keto-3-deoxyheptonate aldolase	• Triosephosphate isomerase
	• Fructose-1,6-bisphosphatase	• 6-phosphofructo-2-kinase 1	• Ribose-5-phosphate isomerase
	• Isocitrate dehydrogenase [NADP] [c]	• Glutamine-dependent NAD(+) synthetase	• Inorganic pyrophosphatase
	• Succinate-semialdehyde dehydrogenase [NADP+]	• Ribose-phosphate pyrophosphokinase 4	• Adenylate kinase [c]
	• Citrate synthase [p]	• ATP-dependent permease AUS1	• Glutamate decarboxylase
	• Isocitrate dehydrogenase [NAD] subunit 1 [m]	• Fructose-2,6-bisphosphatase	• 4-aminobutyrate aminotransferase
	• Pyruvate kinase 2	• Nicotinate-nucleotide pyrophosphorylase [carboxylating]	• Tricarboxylate transport protein
	• Low-affinity glucose transporter HXT4′	• Squalene monooxygenase	• Prephenate dehydrogenase [NADP+]
			• Tricarboxylate transport protein

**Table 3:** Top scoring enzymes for transcriptional, post-transcriptional and metabolic regulation upon deletion of specific genes.

Mutants	Enzymes showing transcriptional regulation	Enzymes showing post-transcriptional regulation	Enzymes showing metabolic regulation
**Wild type-grr1Δ**	• GMP synthase [glutamine-hydrolyzing]	• ADP, ATP carrier protein 2	• Acetyl-CoA carboxylase malonyltransferase
	• Phosphoribosylformylglycinamidine synthase	• Glycogen [starch] synthase isoform 1	• [Acyl-carrier-protein]
	• Dihydroorotase	• AMP deaminase'	• [Acyl-carrier-protein] acetyltransferase
	• Imidazole glycerol phosphate synthase hisHF	• Sugar transporter STL1	• Grouped fatty acid synthesis [c]
	• Adenylosuccinate lyase	• Phosphofructokinase 2	
	• Pantoate–beta-alanine ligase	• D-3-phosphoglycerate dehydrogenase 2	
	• Inorganic phosphate transporter	• Probable 6-phosphogluconolactonase 4	
	• Threonine dehydratase [m]	• Glycerol-3-phosphate dehydrogenase [NAD+] 1	
	• ATP phosphoribosyltransferase	• Alcohol dehydrogenase 4	
	• Histidinol-phosphate aminotransferase	• Xanthine phosphoribosyltransferase 1	
**Wild type-hxk2Δ**	• Phosphoribosylformylglycinamidine synthase	• Nucleoside diphosphate kinase	• Mannose-6-phosphate isomerase
	• Pyruvate decarboxylase isozyme 3	• Cytochrome b-c1 complex subunit Rieske [m]	• Acetyl-CoA carboxylase
	• Phosphoglycerate mutase 2	• Homocysteine S-methyltransferase 1	• [Acyl-carrier-protein] malonyltransferase
	• Alcohol dehydrogenase 5	• ATP-NADH kinase	• [Acyl-carrier-protein] acetyltransferase
	• Imidazole glycerol phosphate synthase hisHF	• 1,4-alpha-glucan-branching enzyme	• Grouped fatty acid synthesis [c]
	• Dihydroorotase'	• NAD-dependent malic enzyme [m]	
	• Hexokinase-2	• Pyrroline-5-carboxylate reductase	
	• Phosphoglycerate mutase 3	• Galactose-1-phosphate uridylyltransferase	
	• Phosphofructokinase 1		
	• Adenylosuccinate lyase		
**Wild Type-mig1Δ**	• Probable 6-phosphogluconolactonase 1	• ADP-sulfurylase	• Mannose-6-phosphate isomerase
	• Orotidine 5′-phosphate decarboxylase	• Purine nucleoside phosphorylase	• Mannose-1-phosphate guanyltransferase
	• Transketolase 2	• 3′,5′-cyclic-nucleotide phosphodiesterase 2	• Acetyl-CoA carboxylase
	• Chorismate mutase	• Phosphoserine phosphatase	• Grouped fatty acid synthesis [c]
	• Prephenate dehydratase'	• Cytidine deaminase	
	• Amidophosphoribosyltransferase	• 1,3-beta-D-glucan-UDP glucosyltransferase	
	• Phosphatidate cytidylyltransferase	• Vacuolar acid trehalase	
	• Diacylglycerol pyrophosphate phosphatase 1	• Low-affinity glucose transporter HXT1	
	• Inositol-3-phosphate synthase	• Trehalose-phosphatase	
**Wild Type-mig1Δmig2Δ**	• Branched-chain-amino-acid aminotransferase [c]	• Cystathionine beta-synthase	• Phosphomannomutase
	• Phosphoribosylformylglycinamidine synthase	• Isocitrate lyase	• Mannose-1-phosphate guanyltransferase
	• Pantothenate kinase	• Mitochondrial dicarboxylate transporter	• Acetyl-CoA carboxylase
	• GMP synthase [glutamine-hydrolyzing]	• High-affinity glucose transporter HXT2	• Grouped fatty acid synthesis [c]
	• Imidazole glycerol phosphate synthase hisHF	• Uridylate kinase	
	• 2-isopropylmalate synthase	• Nucleoside diphosphate kinase	
		• Cytidine deaminase	
		• Potassium-activated aldehyde dehydrogenase [m]	
		• Acetyl-coenzyme A synthetase 2	
		• Pyrroline-5-carboxylate reductase	
**Wild Type-gdh1Δ**		• Malate synthase 2 [g]	• Glycerol uptake/efflux facilitator protein
		• Sugar transporter STL1	
		• 1,3-beta-glucan synthase component FKS3	
		• High-affinity glucose transporter HXT2	
		• S-(hydroxymethyl)glutathione dehydrogenase	
		• Methylenetetrahydrofolate dehydrogenase [NAD+]	
		• Acetate transport via proton symport	
		• Homoaconitase [m]	
		• Homoisocitrate dehydrogenase [m]	
		• Glutamate 5-kinase	

The method to identify the significance of flux changes relies on a set of measured external fluxes, and in some cases strains that don't show significant changes in external fluxes have changes in internal fluxes [18]. These changes cannot be identified with our method, and our estimations of the significance of flux changes can therefore be seen as conservative estimates. The lists of transcriptionally and metabolically regulated reactions are therefore more reliable than the list of post-transcriptionally regulated reactions (in which some fluxes may be changed in reality but their change pass undetected).

The reactions showing transcriptional regulation form a set of putative targets where enzyme over-expression or down regulation will influence the flux through these reactions. The reactions showing metabolic regulation points to parts of the metabolism where the pools of metabolites are possibly increasing or decreasing in connection with transcriptional changes and hereby counteracting possible changes in enzyme concentration as a result of transcriptional changes. This knowledge can be used to identify whether one should target changes in enzyme concentration (v_max_ changes), e.g. through over-expression, or changes in enzyme affinity (K_m_ changes), e.g. through expression of heterologous enzymes, in order to alter the fluxes.

#### Effects of different carbon sources

In the glucose to maltose transition, only two enzymes showed transcriptional change correlated with their flux. The α-glucosidase MAL32, responsible for the breakdown of maltose into glucose was up-regulated and the glucose transporter HXT4 was down-regulated. The metabolic adjustment in terms of fluxes was minimal and only enzymes directly related with substrate uptake and utilization were detected. The changes in gene expression were also low and only 11 metabolic enzymes were significantly perturbed (without significant flux changes).

The glucose to ethanol and the glucose to acetate transitions showed widespread flux and expression changes, and they are therefore more interesting study cases. In the glucose-ethanol transition 19 enzymes showed transcriptional regulation and 22 other enzymes changed in expression but not in flux. For the glucose-acetate transition the same numbers were 33 and 23 respectively. We can see that about one half of the genes that changed in transcription level also showed significant flux changes, this proportion is higher than in the case of deletion mutants (as it will be discussed later). Among the enzymes showing transcriptional regulation, 14 were shared between the glucose-ethanol and glucose-acetate transitions. Interestingly, no overlap was found between the sets of enzymes that don't change in flux. Metabolic regulation was observed in 21 reactions for each case, among which 8 overlap.

The enzymes showing transcriptional regulation clearly show a down regulation of enzymes involved in glucose uptake and utilization (e.g. Glucose transporter HXT4 or Hexokinase 2) and the up-regulation of enzymes involved in the gluconeogenesis (e.g. Fructose-1,6-biphosphatase) and the TCA cycle (e.g. Succinate dehydrogenase or Citrate synthase). The AcCoA synthetase 2, responsible for supplying AcCoA to the TCA cycle is also transcriptionally up-regulated as well as the ATP synthetase (involved in the respiratory chain) and the external NADH-ubiquinone oxidoreductase 2, which supplies the necessary NAD^+^ to oxidize ethanol or acetate in the cytoplasm and maintain the redox balance in the cell. Isocitrate lyase, a key component of the glyoxylate cycle, is also transcriptionally up regulated and this allows for net formation of malic acid that can further be converted to phopshoenolpyruvate (via oxaloacetate) that fuels the gluconeogenesis. All these changes in fluxes are consistent with general knowledge about the changes in metabolism from glucose to C2 carbon sources like ethanol and acetate, but what is interesting to see is that not all the reactions associated with these flux changes are transcriptionally regulated, but the cell have selected a few key reactions to regulate at the transcriptional level and these are identified using our method.

In order to make a deeper analysis, we performed an enrichment test to compare the transcription factors involved in the expression of the enzymes showing transcriptional regulation and the enzymes showing changes in expression but not in flux. We found three transcription factors that were strongly overrepresented in the metabolic genes showing transcriptional regulation. In the glucose-ethanol transition, the transcription factors Gcr1 and Gcr2 both appeared in 11 transcriptionally regulated genes and in none of the other genes, whereas the transcription factor Hap4 appeared in 11 transcriptionally regulated genes and 5 of the other regulated genes. For the glucose-acetate transition these numbers were 15-0, 11-0 and 15-0 for the same transcription factors. This means that certain transcription factors are especially involved in the transcriptional regulation of metabolic fluxes (the same kind of enrichment was observed in the deletion mutants, as will be discussed later), and to our knowledge this has not been previously reported. It basically implies that there is global regulation of major flux alterations, which is similar to what has experimentally been shown to be the case for galactose metabolism [19].

The top scoring metabolically regulated reactions, both for glucose-ethanol and glucose-acetate, are the Fructose biphosphate aldolase and the Triosephosphate isomerase. These reactions are known to operate close to the equilibrium and are therefore very sensitive to changes in the metabolic pools, which is consistent with metabolic regulation of the fluxes. In the considered cases the direction of these reactions is inverted. This can only be explained by a decrease in the fructose-1,6-diphosphate pool and an increase in the glyceraldehyde-3-phosphate and dihydroxyacetone pools. This hypothesis is supported by the fact that in chemostat cultures, there was not found any correlation between the glycolytic flux and the expression of the genes encoding these two enzymes [20].

**Figure 4:**
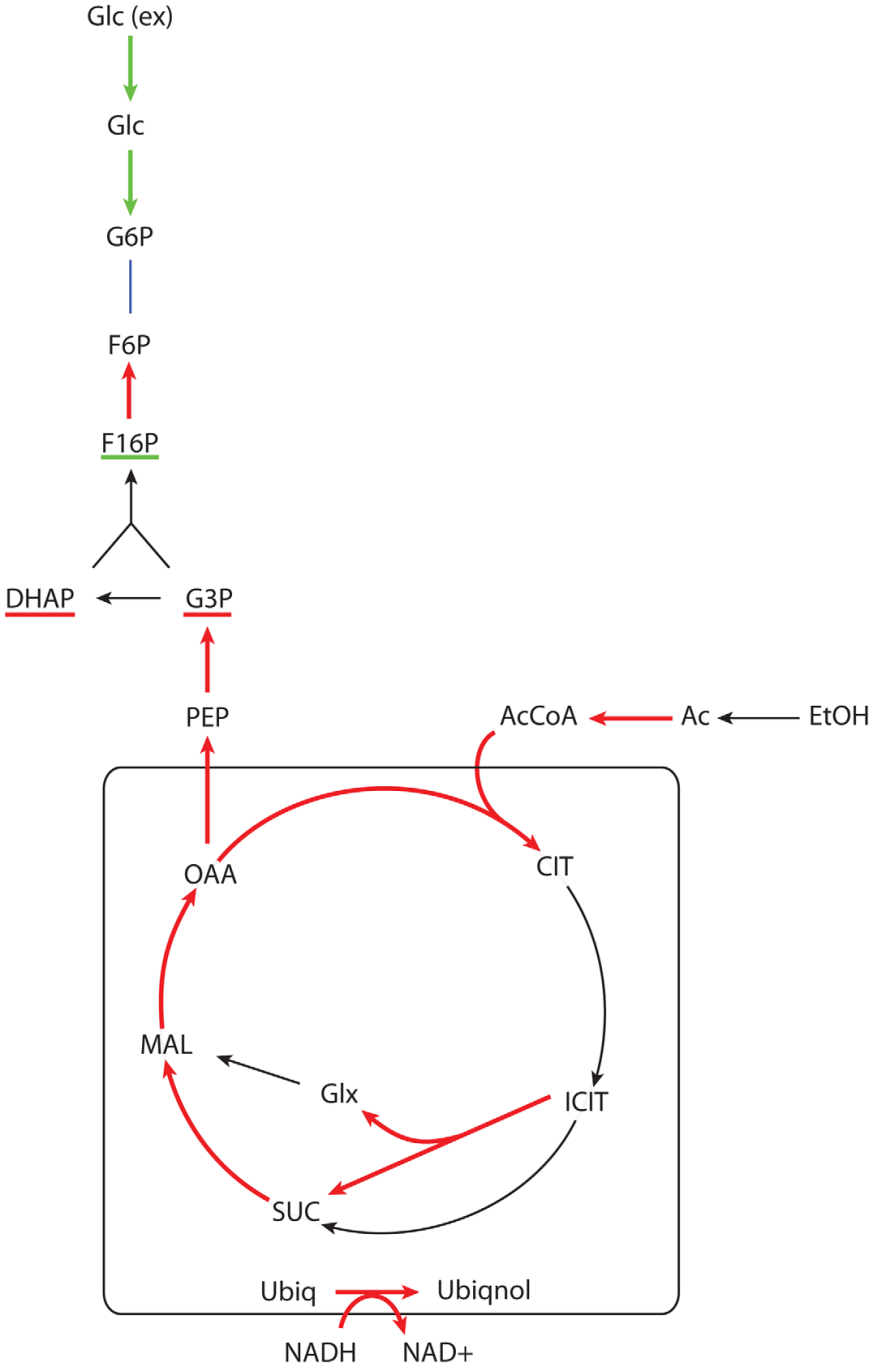
Main reactions showing transcriptional up (red) or down (green) regulation associated with the glucose-ethanol shift. The underlined metabolite pools are those that are expected to increase (red) or decrease (green) according to the observed metabolic regulation.

The results for the glucose-ethanol change are summarized in Fig. 4.

#### Effects of gene deletions

As mentioned above several different deletion strains were evaluated and the number of enzymatic reactions showing transcriptional regulation was 26 for the grr1Δ strain, 25 for the hxk2Δ strain, 11 for the mig1Δ strain, 8 for the mig1Δmig2Δ strain and 0 for the gdh1Δ strain. The reactions showing post-transcriptional regulation were 73, 70, 46, 36 and 89 for the same strains, respectively. These numbers clearly show, that in contrast to growth on different carbon sources, most of the transcriptional changes do not result in correlated changes in metabolic fluxes. This indicates that many transcriptional changes are indeed happening in order to minimize the metabolic adjustment resulting from a gene deletion, and the most extreme case is the gdh1Δ strain, where no transcriptional changes seem to be correlated to flux changes. This behaviour is consistent with the MOMA (Minimization of Metabolic Adjustment) hypothesis [21].

There is a substantial overlap between the strains grr1Δ and hxk2Δ, 15 transcriptionally regulated reactions were shared between both strains and 28 post-transcriptionally regulated reactions were also shared. An enrichment test was performed in order to find transcription factors regulating the enzymes showing transcriptional regulation. For the grr1Δ, the most significantly enriched transcription factors were Pho2 (which regulates the expression of 10 of the 26 enzymes with transcriptional regulation and only 6 of the 73 enzymes showing post-transcriptional regulation) and Bas1 (which regulates 10 out of 26 and 7 out of 73 enzymes in the mentioned groups). For the hxk2Δ strain the most significant transcription factor enrichment was found for the factors Pho2 (with 7 and 5 enzymes in each of the groups) and Bas1 (with 7 and 6 enzymes in each of the groups).

Pho2 and Bas1 are partner proteins that regulate the transcription of genes involved in purine and histidine biosynthesis [22]. It is possible that the slower growth rate observed in grr1Δ and hxk2Δ with respect to the wild type is due to a down-regulation of purine and histidine biosynthesis resulting from lower activities of Pho2 or Bas1.

Strains grr1Δ and hxk2Δ show specific growth rates of 0.23 and 0.22 h^−1^ respectively [16], however the biomass yields were 0.09 g-DW g^−1^ and 0.2 g-DW g^−1^. The specific glucose uptake rate for the hxk2Δ strain is significantly lower than for the grr1Δ strain as well as for the reference strain. This is associated with the observation that the glycolytic flux in the hxk2Δ strain shows transcriptional down-regulation of five enzymes. In the upper glycolysis, the Hexokinase 2 has been deleted and the Phosphofructokinase 1 is down-regulated. The Phosphofructokinase 1 was also strongly down-regulated in the grr1Δ strain but the decrease in flux was not as large as in the hxk2Δ strain. In the lower glycolysis of the hxk2Δ, all the three iso-enzymes of Phosphoglycerate mutase were down-regulated as well as the phosphoglycerate kinase. No down-regulation for these enzymes was seen in the grr1Δ strain. The glycerol-3-phosphate dehydrogenase has two iso-enzymes. The first of those isoenzymes was up-regulated in the grr1Δ and the hxk2Δ strains; however its expected flux decreased in both cases (more significantly in hxk2Δ). The second iso-enzyme did not show important changes in grr1Δ but was down-regulated in hxk2Δ. This is not the only case in which different iso-enzymes show different regulatory patterns, and in these cases our method for having flux estimations independent from transcriptome analysis is particularly useful.

**Figure 5:**
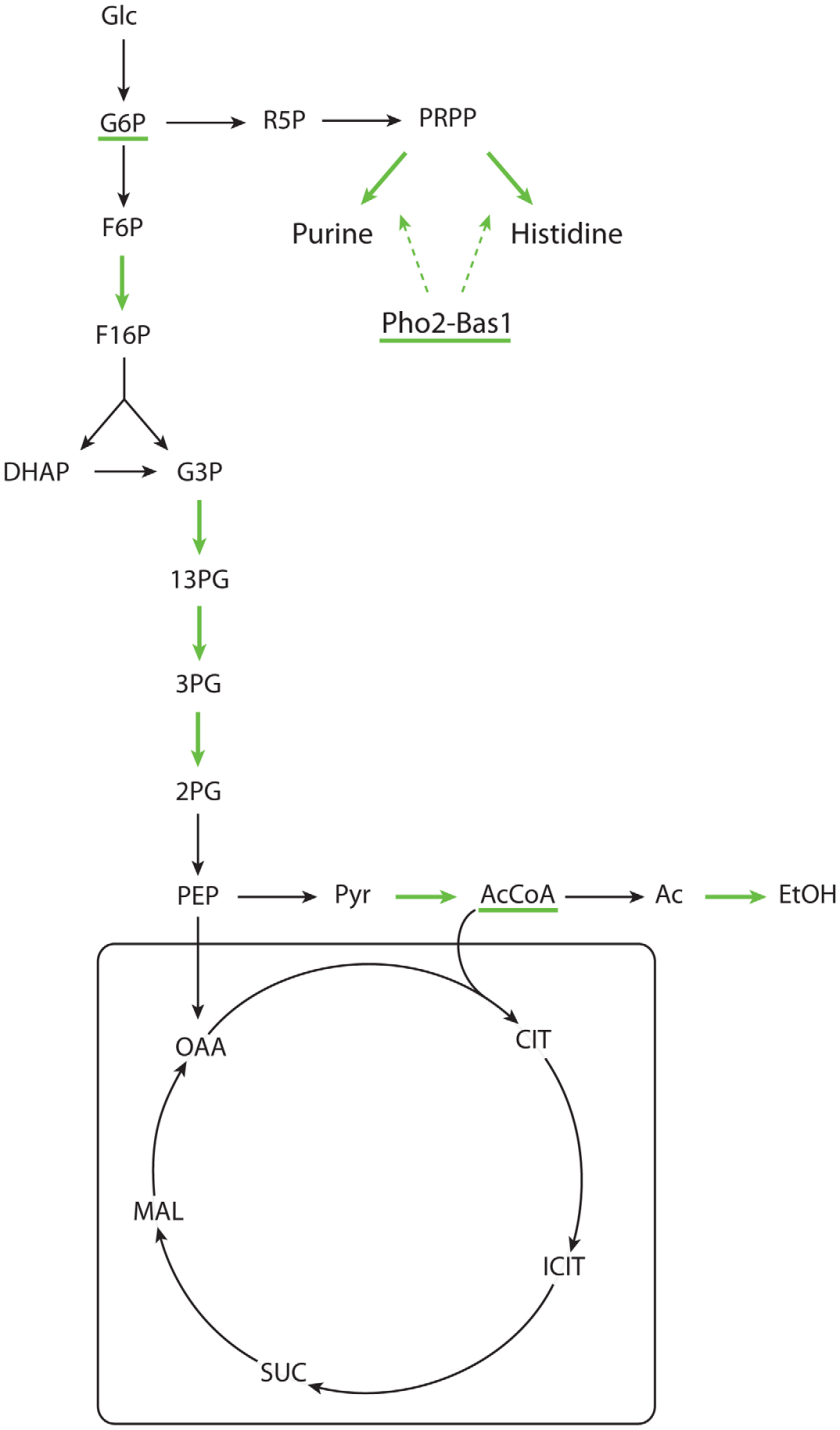
Main reactions showing transcriptional up (red) or down (green) regulation associated with the deletion of HXK2. The underlined metabolite pools are expected to increase (red) or decrease (green) according to the observed metabolic regulation. The transcription factors controlling the down-regulated pathways are also underlined in green.

The strain hxk2Δ showed a strong decrease in ethanol production compared to grr1Δ. All the alcohol dehydrogenase iso-enzymes were down-regulated in a similar way in both strains. The explanation of the differences in flux towards ethanol should be found in the pyruvate decarboxylase. Pyruvate decarboxylase 3 was strongly down-regulated in both strains, however, pyruvate decarboxylase 2 was up-regulated. This up-regulation was much more significant in grr1Δ, which could explain a higher flux from pyruvate to AcCoA in this strain. The results for the hxk2Δ mutant are summarized in Fig. 5.

The mig1Δ mutant shows a higher specific growth rate than the wild type. In general it is transcriptionally very similar to the wild type [16]. An enrichment test in transcription factors between transcriptional regulated and post-transcriptional regulated reactions was performed and the factor Sfp1 was found. This factor is known to regulate ribosome production and is nutrient sensitive [23]. This could mean that the deletion of *MIG1* activates a response against starvation that results in an increased specific growth rate. Among the transcriptionally regulated reactions, a slight down-regulation of the PP pathway is observed together with an up-regulation of several amino-acid production pathways.

In the mig1Δmig2Δ mutant there is a slight decrease in the specific growth rate. All the 8 transcriptionally regulated reactions were down-regulated and belonged to amino-acid biosynthesis pathways. The enrichment test found the factors Cbf1 and Gcn4 (represented in 4 and 1 out of 8 reactions and 5 and 6 out of 36 reactions). Gcn4 is known to regulate amino-acid biosynthetic genes [24] and it seems that the up-regulation of amino-acid biosynthesis due to the deletion of *MIG1* is cancelled by an opposite effect due to *MIG2*.

In all the mutants discussed above, the AcCoA carboxylase and the fatty acid synthesis showed metabolic regulation. This could indicate that in the studied cases the AcCoA pool was the main parameter responsible for adjusting the rate of lipid biosynthesis to match the changes in specific growth rates.

The experiments for the gdh1Δ mutant were performed in chemostat cultures [17] with the same dilution rate. The only observed change in exchange fluxes was a small decrease in glycerol production and only a few significant changes were identified in the metabolic fluxes. However, there were significant transcriptional changes in many metabolic pathways. This again points to the hypothesis that changes in transcription mainly results in altered metabolite levels such that metabolic homeostasis can be maintained. This is supported by metabolome analysis of this mutant, which showed that there were many changes in the metabolite levels [25] and in fact many of these changes were associated with changes also in the transcription of associated enzymes [26]. It is possible that the chemostat conditions, by imposing the same specific growth rate, forced the mutant strain to important transcriptional changes in order to keep the fluxes unchanged.

**Figure 6:**
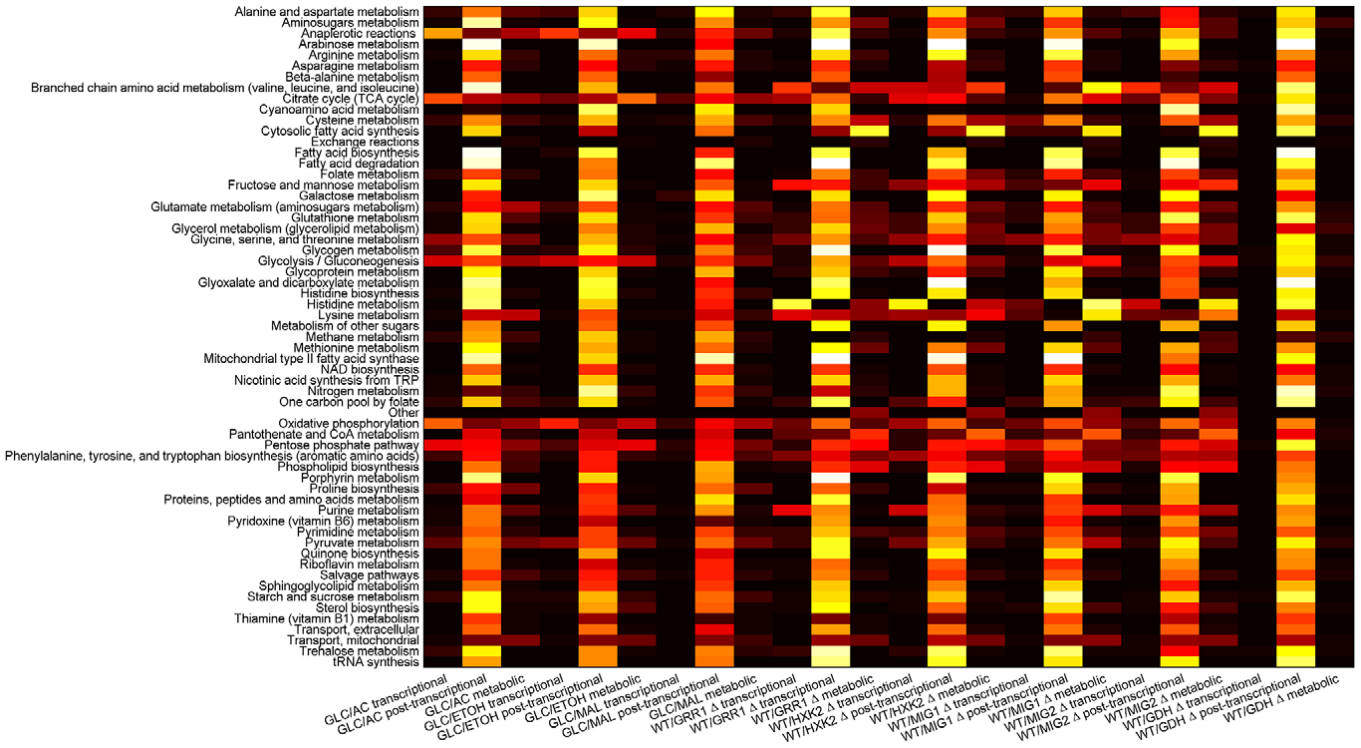
This figure illustrates the extent of transcriptional, post-transcriptional and metabolic regulation observed in different metabolic processes for each of the studied cases. The metabolic processes are defined in the same way as in the iFF708 model. The brightness of the color is proportional to the probability of a reaction in the corresponding process to show transcriptional, post-transcriptional and metabolic regulation respectively. The black correspond to 0 and the white to 1.

In Fig. 6 we aim to provide a global visualization of the changes for all the studied metabolic conditions.

## Methods

### Sampling in the region of feasible solutions

The steady state condition and the irreversibility of some reactions impose limitations on the flux distributions attainable by the cell [18]. The set of feasible solutions can be further constrained by fixing some fluxes to their experimental values. In general, the fluxes most accessible to experimental determination are those corresponding to uptake or secretion rates. After fixing a subset of fluxes, genome scale models still have a large number of degrees of freedom. In this study we used the genome scale model iFF708 for *S. cerevisiae* [27]. Random sampling has previously been performed [7] by enclosing the region of allowed solutions in a parallelepiped with the same dimensions as solution space (the null space of the stoichiometric matrix) and generating random points inside this parallelepiped. The points that lie inside the region of possible solutions are then selected. The COBRA Toolbox [12] uses a Hit and Run algorithm to generate random points in this way. In this work instead of sampling inside the region of allowed solutions we sampled at its corners.

In order to obtain corners in the space of allowed solutions we used the simplex method with a random set of objective functions to be maximized. The maximization of each of these objective functions will give a corner in the space of solutions. The constraints imposed upon each optimization are:
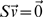
(1)

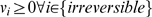
(2)

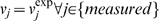
(3)The values of the measured fluxes (***v^exp^***) are different between conditions. This fact changes the shape of the region of feasible solutions between different conditions. *S* is the stoichiometric matrix of the network.

In order to reduce the effects of internal loops we first identified all the reactions that can get involved in loops using the FVA (Flux Variability Analysis) option in the COBRA Toolbox. The reactions that can be involved in loops are unbounded and show the default maximal or minimal value set in the COBRA Toolbox (1000 or −1000). If these bounds were kept, the means and standard deviations for these reactions would be unrealistic [6] and cannot be used for further analysis. In order to reduce the effect of loops, the default maximal and minimal fluxes for the reactions involved in loops, were set to a smaller value in order to reduce the loop effect. In order to select an appropriate value the bounds were increased from 0 in steps of 0.1 until the minimal value that allows obtaining flux distributions consistent with the experimental fluxes is found. These values went from 1 to 15 mmol h^−1^g-DW^−1^ depending on each condition. Also no weights (eq. 4) were assigned to the reactions involved in loops in order to avoid objective functions that maximize the activity of loops.

Random objective functions were generated by selecting random pairs of reactions and assigning them random weights (the reactions involved in loops were excluded from these choices). The weights (*w_i_*) assigned to each reaction were generated by dividing a random number between 0 and 1 by the maximal flux for this reaction obtained using FVA. This normalization was made to account for the different size orders of the different reactions. The objective functions take the form:
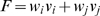
(4)One solution is obtained for each of the objective functions generated.

Our objective is to obtain means and standard deviations for each flux in each of the compared conditions and use them to get a Z-score quantifying the significance of change in each flux between the considered conditions. This score is equal to the difference between the means in each of the conditions divided by the standard deviation of this difference (note that the variance of the difference is the sum of the two variances and the standard deviation its square root).
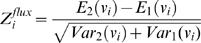
(5)The difference between averages in the numerator follows a normal distribution (according to the central limit theorem) with a standard deviation equal to the standard deviation of the flux (the denominator in eq. (5)) divided by the square root of the number of samples. Therefore, Z itself follows a normal distribution with a standard deviation equal to the inverse of the square root of the number of samples.

The Z score measures the significance of change in terms of standard deviations. If the error in the Z score is lower than 0.15, no information would be lost in terms of classifying a reaction as significantly changed or not. The order of size of a genome-scale model is about 1000 reactions. A reasonable accuracy for the Z-scores would be to expect errors higher than 0.15 on the Z score only for 1 reaction in the whole model. This means a p-value of 0.001. If we want to keep the error on the Z score under 0.15 with a probability of 0.999 we need 500 samples, and this was therefore selected as the sampling number.

### Classification of enzymes according to their changes in flux and expression level

The Z-scores can be transformed into probabilities of change by using the cumulative Gaussian distribution. Once we have Z-scores for the significance of flux changes and Z-scores for the significance of gene-expression changes we can obtain probabilities of having correlated expression and flux changes for each enzyme.

An increase in enzyme expression can result in an increase of flux (transcriptional regulation). In order to evaluate the probability for a reaction of being transcriptionally regulated we multiply the probability of its enzyme level changing by the probability of its flux changing in the same direction (obtained using the cumulative normal distribution).
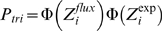
(6)

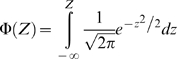
(7)If there is a decrease in expression and a decrease in flux, both Z-scores are negative and we will use the absolute values of the Zs in eq. (6). If there is an increase in expression and a negative flux becomes more negative, we will use the absolute value of the Z-score for the flux change. If the direction of the flux changes between conditions, this change must be driven by the metabolic concentrations and no by transcriptional regulation, therefore a P_tri_ of zero is assigned by default.

In the same way as in eq. (6) we can define probabilities for the expression level changing and for the flux not changing (post transcriptional regulation).
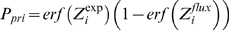
(8)

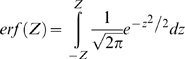
(9)Now we use the error function because we want to evaluate the probability of change in any direction. The absolute value of Z is used in all the cases.

The probability of a change in flux but not in transcription (metabolic regulation) can be obtained for each reaction as follows:
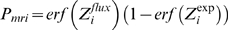
(10)Each of these three probabilities can be associated to each enzyme in the metabolic network.

Table 4 summarizes the criteria to assign each type of regulation.

**Table 4:** Decision table to assign transcriptional regulation (TR), post transcriptional regulation (PR) or metabolic regulation (MR) to the different enzymes depending on the observed up-regulation (+), down-regulation (−) or lack of change ( = ) of flux and gene expression.

Exp\Flux	+	−	=
+	TR	MR	PR
−	MR	TR	PR
=	MR	MR	

## References

[1] Patil KR, Nielsen J (2005). Uncovering transcriptional regulation of metabolism by using metabolic network topology.. Proc Natl Acad Sci U S A.

[2] Gygi SP, Rochon Y, Franza BR, Aebersold R (1999). Correlation between protein and mRNA abundance in yeast.. Mol Cell Biol.

[3] Yang C, Hua Q, Shimizu K (2002). Integration of the information from gene expression and metabolic fluxes for the analysis of the regulatory mechanisms in Synechocystis.. Appl Microbiol Biotechnol.

[4] Moxley JF, Jewett MC, Antoniewicz MR, Villas-Boas SG, Alper H (2009). Linking high-resolution metabolic flux phenotypes and transcriptional regulation in yeast modulated by the global regulator Gcn4p.. Proc Natl Acad Sci U S A.

[5] Fong SS, Nanchen A, Palsson BO, Sauer U (2006). Latent pathway activation and increased pathway capacity enable Escherichia coli adaptation to loss of key metabolic enzymes.. J Biol Chem.

[6] Mo ML, Palsson BO, Herrgård MJ (2009). Connecting extracellular metabolomic measurements to intracellular flux states in yeast.. BMC systems biology.

[7] Schellenberger J, Palsson BØ (2008). Use of randomized sampling for analysis of metabolic networks.. J Biol Chem.

[8] Almaas E, Kovács B, Vicsek T, Oltvai ZN, Barabási AL (2004). Global organization of the metabolic fluxes in the bacterium *Escherichia coli*.. Nature.

[9] Papin JA, Reed JL, Palsson BØ (2004). Hierarchical thinking in network biology: the unbiased modularization of biochemical networks.. Trends Biochem Sci.

[10] Jamshidi N, Palsson BO (2007). Investigating the metabolic capabilities of Mycobacterium tuberculosis H37Rv using the in silico strain iNJ661 and proposing alternative drug targets.. BMC Syst Biol.

[11] Thiele I, Price ND, Vo TD, Palsson BØ (2005). Candidate metabolic network states in human mitochondria: Impact of diabetes, ischemia, and diet.. Journal of Biological Chemistry.

[12] Becker SA, Feist AM, Mo ML, Hannum G, Palsson BØ (2007). Quantitative prediction of cellular metabolism with constraint-based models: the COBRA toolbox.. Nat Prot.

[13] Lovasz L (1999). Hit and Run mixes fast.. Math Program.

[14] Jouhten P, Rintala E, Huuskonen A, Tamminen A, Toivari M (2008). Oxygen dependence of metabolic fluxes and energy generation of Saccharomyces cerevisiae CEN.PKI I3-IA.. BMC Sys Biol.

[15] Daran-Lapujade P, Jansen MLA, Daran JM, van Gulik W, de Winde JH (2004). Role of transcriptional regulation in controlling fluxes in central carbon metabolism of Saccharomyces cerevisiae.. J Biol Chem.

[16] Westergaard SL, Oliveira AP, Bro C, Olsson L, Nielsen J (2007). A systems biology approach to study glucose repression in the yeast Saccharomyces cerevisiae.. Biotechnol Bioeng.

[17] Bro C, Regenberg B, Nielsen J (2004). Genome-wide transcriptional response of a Saccharomyces cerevisiae strain with an altered redox metabolism.. Biotechnol Bioeng.

[18] Price ND, Reed JL, Palsson BØ (2004). Genome scale models of microbian cells: Evaluating the consequences of constraints.. Nat Rev Microbiol.

[19] Ostergaard S, Walløe KO, Gomes CSG, Olsson L, Nielsen J (2001). The impact of GAL6, GAL80 and MIG1 on glucose control of the GAL system in Saccharomyces cerevisiae.. FEMS Yeast Res.

[20] Daran-Lapujade P, Rossell S, van Gulik WM, Luttik MAH, de Groot MJL (2007). The fluxes through glycolytic enzymes in Saccharomyces cerevisiae are predominantly regulated at posttranscriptional levels.. Proc Natl Acad Sci U S A.

[21] Segre D, Vitkup D, Church GM (2002). Analysis of optimality in natural and perturbed metabolic networks.. Proc Natl Acad Sci U S A.

[22] Bhoite LT, Allen JM, Garcia E, Thomas LR, Gregory ID (2002). Mutations in the Pho2 (Bas2) transcription factor that differentially affect activation with its partner proteins Bas1, Pho4, and Swi5.. J Biol Chem.

[23] Marion RM, Regev A, Segal E, Barash Y, Koller D (2004). Sfp1 is a stress- and nutrient-sensitive regulator of ribosomal protein gene expression.. Proc Natl Acad Sci U S.

[24] Hope IA, Struhl K (1987). Gcn4, a eukaryotic transcriptional activator protein, binds as a dimer to target DNA.. EMBO J.

[25] Villas-Bôas SG, Moxley JF, Åkesson M, Stephanopoulos G, Nielsen J (2005). High throughput metabolic state analysis: the missing link in integrated functional genomics of yeasts.. Biochem J.

[26] Cakir T, Patil KR, Önsan ZI, Ülgen KÖ, Kirdar B (2006). Integration of metabolome data with metabolic networks reveals reporter reactions.. Mol Syst Biol.

[27] Förster J, Famili I, Fu P, Palsson BØ, Nielsen J (2003). Genome-scale reconstruction of the Saccharomyces cerevisiae metabolic network.. Genome Res.

